# Electroacupuncture alleviates the symptom of depression in mice by regulating the cGAS-STING-NLRP3 signaling

**DOI:** 10.18632/aging.205596

**Published:** 2024-04-19

**Authors:** Shiyun Chen, Jingjing Li, Luda Yan, Xianhao Zhang, Jiesi Huang, Peng Zhou

**Affiliations:** 1 Department of Acupuncture, Shenzhen Bao’an Traditional Chinese Medicine Hospital, Bao’an, Shenzhen, Guangdong Province, China

**Keywords:** depression, electroacupuncture, cGAS-STING-NLRP3 signaling, neuroinflammation

## Abstract

Purpose: To investigate the therapeutic effect of electroacupuncture (EA) on chronic and unpredictable mild stress (CUMS)-induced depression in mice and the underlying mechanism.

Methods: Male C57BL/6 mice were randomly divided into 6 groups: Control, CUMS, CUMS+EA-placebo, CUMS+EA, CUMS+ ad-NC, CUMS+ ad-cGAS-shRNA. CUMS was utilized to establish the depression model in mice. The behavioral changes were determined by the forced swimming, open field, and sucrose preference experiments. The pathological changes in the hippocampus tissue were evaluated by HE staining. The release of TNF-α, IL-1β, IL-6, 5-HT, and NE in the hippocampus tissue was determined by ELISA. IBA-1 expression detected by the immunofluorescence was used to represent the activity of microglia. Western blot and RT-PCR were utilized to measure the expression of Bax, bcl-2, cGAS, STING, TBK1, IRF3, and NLRP3.

Results: The depression behavior in CUMS mice was significantly alleviated by the treatment of EA and cGAS-shRNA, accompanied by ameliorated hippocampus pathological changes, declined production of TNF-α, IL-1β, and IL-6, elevated secretion of 5-HT and NE, and inhibition on the activity of microglia. Furthermore, significantly elevated expression level of Bax, cGAS, STING, TBK1, IRF3, and NLRP3 and declined expression level of bcl-2 were observed in the CUMS+EA and CUMS+ ad-cGAS-shRNA groups.

Conclusions: EA significantly mitigated the symptom of depression in mice, which was closely associated with the repressed neuroinflammation, increased monoamine concentration, inactivated microglia, and inhibited cGAS-STING-NLRP3 signaling.

## INTRODUCTION

Depression is a global mental health problem with high incidence, recurrence, disability and suicide risk. Depression patients are a high-risk population for suicide. According to the statistics of the World Health Organization, about 350 million people in the world are suffering from depression [1]. The pathogenesis of depression is complex, and due to the lack of accurate and effective therapeutic targets, the efficacy of modern medicine in treating depression is poor [2]. Therefore, it is particularly important to investigate the treatment of depression.

Depression belongs to the category of “depression syndrome” in traditional Chinese medicine, which is mainly caused by emotional disorders and the inability of liver Qi-blood to reach a comfortable condition. It has been recorded that ancient doctors used acupuncture to treat depression syndrome. According to the recordation in Compendium of Acupuncture and Moxibustion, acupuncture and moxibustion regulate collaterals and Qi-blood in treating depression.

Acupuncture is a method of preventing and treating diseases using needle insertion and moxibustion. Needle insertion involves inserting a metal needle into a specific acupoint on the human body, and using techniques to adjust the flow of Qi and blood [3]. EA therapy is a new intervention method that combines traditional needle insertion with micro-bioelectric current to provide continuous positive stimulation. Numerous studies have been conducted on the mechanism of the effect of EA intervention on depression, and the efficacy of EA in treating depression has gradually been verified [4, 5]. The study by She et al. [6] showed that a certain improvement effect on the depressed rat model was achieved using EA intervention (Baihui point and Yintang point), which was closely associated with the plasticity of hippocampal synapses. Lee et al. reported that certain environmental effects on the mouse model of depression were achieved using EA intervention (KI10·LR8·LU8·LR4), which was possibly related to central nervous activity and regulation of serotonin receptors [7]. Yang et al. confirmed the therapeutic effect of EA (Baihui point and Yintang point) in the depression rat model [8]. EA has a therapeutic effect on animal models of depression, but its specific molecular mechanism remains unclear.

Numerous studies have reported the association among depression, stress and neuroinflammation. Damage-associated molecular patterns (DAMPs) and central nervous system inflammation triggered by chronic stress will be induced by the activated microglia, which can be promoted by NLRP3 inflammasome by releasing IL-1β and inducing an inflammatory cascade. The clinical study has reported that elevated levels of pro-inflammatory cytokines IL-1β, IL-6, and TNF-α are observed in depression patients, accompanied by the activation of microglia in the central nervous system [9]. Fingolimod is reported to regulate NLRP3 and microglia activation, leading to chronic and unpredictable depression-like behavior induced by mild stress [10]. Hesperidin improves chronic and unpredictable mild stress-induced depression by inhibiting inflammation of microglia and regulating NLRP3 validation signaling pathway [11]. The total alkaloids of Fibraurea recisa activate the BDNF-MEK/ERK neuroprotective pathway and inhibit the NLRP3/caspase-1 inflammatory signaling pathway, alleviating CUMS-induced depression behavior in mice [12]. Chen et al. investigated the anti-depressive effect of Bupleurum through non-targeted metabolomics and targeted quantitative analysis, and the results showed that the inhibition of oxidative stress and the regulation of neuroinflammation in the cortex are involved in the synergistic anti-depressive effect of Bupleurum [13]. These studies suggest that neuroinflammation is a factor that cannot be ignored in the development of depression.

The CGAS-STING axis is composed of cyclic-GMP-AMP synthase (cGAS) and cyclic GMP-AMP receptor stimulator of interferon genes (STING), and plays an important role in a variety of diseases, including malignant tumors, DNA damage, and inflammation-related diseases [14, 15]. CGAS is the main DNA sensor of innate immune response, which enhances the synthesis of cGAMP by binding to double-stranded cytoplasmic DNA from viral infection, damaged nucleus or damaged mitochondria. CGAMP binds and promotes the activation of STING [16], which then migrates from the endoplasmic reticulum to the Golgi apparatus and activates tank-binding kinase 1 (TBK1). Interferon regulator 3 (IRF3) will be activated by the phosphorylated TBK1 [17]. In rats with pulmonary ischemia-reperfusion, the cGAS-STING signaling pathway is activated. Blocking the cGAS-STING signaling pathway can inhibit endoplasmic reticulum stress-induced apoptosis, reduce inflammation and oxidative stress, and slow down pulmonary edema and pathological damage in I/R rats [18]. In hypertension-induced myocardial injury, cGAS participates in neuroinflammation by impairing autophagy flux and inducing the pro-inflammatory phenotype of microglia, leading to sympathetic overactivation in hypertension and further causing myocardial injury in hypertension [19]. Furthermore, the NLRP3 and downstream inflammatory signaling pathways can be activated by the CGAS-STING signaling [20, 21]. It is reported that aging-related cerebral infarction is alleviated by EA through downregulating the NLRP3/Caspase-1 signaling [22]. Currently, the regulatory relationship between CGAS-STing-NLRP3 signaling axis and depression has not been clarified. Therefore, the present study proposed a hypothesis that the potential molecular mechanism of EA in the treatment of depression in rats is associated with the regulation of the CGAS-STING-NLRP3 signal axis.

## MATERIALS AND METHODS

### Animal experiments

36 C57BL/6 male 6-8 weeks old mice were purchased from Hunan Slaike Jingda Laboratory Animal Co. LTD, and animals were maintained at a temperature of 18° C-26° C and humidity of 30%-70%. They were divided into 6 groups: Control, CUMS, CUMS+EA-placebo, CUMS+EA, CUMS+ad-NC, CUMS+ad-cGAS-shRNA. A mouse model of depression was established by alternate stimulation of mice once a day in various unpredictable random ways for 28 days. Stress sources included fasting for 48h, water restriction for 24h, tail clipping for 60s, heat stimulation at 45° C for 5min, tail suspension for 5min, day and night reversal, and cold stimulation at 4° C for 5min. On average, each stimulation was performed 4 times. Animals were raised in single cages. The EA treatment was performed according to the method described by Lee et al. [7]. The specific operation was to fix the mouse in a cotton mouse sleeve that could extend its limbs and keep still for 5 min, allowing the mouse to adapt before performing EA. The EA was conducted on the 14th day of modeling, using a disposable sterile stainless steel acupuncture needle (diameter 0.18 mm; length 8 mm), acupoints were KI10·LR8·LU8·LR4, needle depth was 3-4 mm, needle rotated at a speed of two rotations per second for 30 s, and then immediately removed. EA treatment was conducted every day for 2 weeks. In the CUMS+EA-placebo group, acupoints were LU8·LR4·HT8·LR2, with the same acupoint selection and treatment time as the EA treatment groups. Adenovirus intervention was performed on the 21st day of mouse modeling by injecting adenovirus containing shRNA against cGAS or shRNA-NC into the lateral ventricle. The large-scale packaging, purification, and titer determination of Adv-cGAS-shRNA required for this study were completed by Jiangxi Zhonghong Boyuan Biotechnology Co. Ltd (China). The enhanced green fluorescent protein (Adv-EGFP) packaged with adenovirus vector was used as a blank control virus, and the determined virus titer was approximately 2.0×10^11^ PFU/mL.

### Open field test

On the second day following 28-day CUMS stimulation, mice were placed into the mouse spontaneous activity kit for 10 min, and the spontaneous activity of the mice within 30 min was recorded. The experiment was carried out between 9:00 and 18:00 in a quiet room at 25° C.

### Forced swimming test

On the second day following 28-day CUMS stimulation, mice were placed in a round water tank with a diameter of 100 cm and a height of 40 cm, with a water depth of 30 cm and a water temperature of (22±3)° C, so that mice could not reach the bottom of the tank upright and could not jump out of the tank. Mice were timed for 5min, and the immobility time (the time of immobility and stepping on fake water) was observed and recorded within 5min.

### Sucrose preference experiment

The sucrose preference experiment was conducted according methods described previously [23]. Before the test, two identical water bottles were placed in each cage, and the mice were trained to drink from the two water bottles. During the first 24 h, both bottles contained the same amount (100 ml) of 1% sucrose solution, and in the second 24 h, one bottle contained (100 ml) 1% sucrose solution, and the other bottle contained (100 ml) water. The mice were placed on a fasting diet for food and water for a period of 20 h after adaptive training. On the next day, the experiment began. One bottle of 100 ml of sucrose water and one 100-ml bottle of water were randomly placed in each cage, removed after 1 h, and reweighed to record the volumes of fluid consumed. Sugar preference value = sugar consumption/(sugar consumption + water consumption) × 100%.

### Animal sacrificing and tissue collection

After the behavioral experiment, the mice were sacrificed by an overdose of anesthesia with intraperitoneal injection of pentobarbital sodium. After anesthetizing the mouse, blood was collected from the abdominal aorta using a non-anticoagulant tube for clinical use. After centrifuging at 3000 r/min for 10min with a radius of 3 cm, the supernatant was collected and stored at -80° C. Half of the mice in each group were sacrificed and their hippocampal tissue was fixed in 4% paraformaldehyde solution for 48 h for HE staining. The other half of the hippocampal tissue was immediately stored in liquid nitrogen for subsequent qPCR, Western Blot, and ELISA detection.

### HE staining

Hippocampus tissues of each animal were collected, which were fixed utilizing 4% paraformaldehyde, followed by gradient dehydration using different concentrations of ethanol solution. After embedding with paraffin, tissues were cut into 4-6 μm sections, which were then dewaxed and hydrated. Subsequently, slides were dyed in hematoxylin aqueous solution for 3min, followed by being re-dyed for 15 s. Sections were then dyed with eosin for 4 min, followed by taking images using the inverted microscope (CX41, OLYMPUS, Tokyo, Japan).

### Real time PCR

In brief, RNAs were extracted from hippocampus tissues utilizing the TRIzol reagent, which were further transcribed into cDNA with a HiFiScript cDNA Synthesis Kit (CW2569M, CWBIO, Beijing, China). The PCR reaction was conducted using the SYBR Green Real-time PCR Master Mix (TOYOBO, Tokyo, Japan), followed by determining the expression of genes with the 2^−ΔΔCt^ method after normalization with the expression of GAPDH. The sequences of primers were listed in Table 1.

**Table 1:** The sequences of primers.

**Genes**	**Sequences (5’-3’)**
β-actin F	AGGGAAATCGTGCGTGAC
β-actin R	CATACCCAAGAAGGAAGGCT
cGAS F	GTCGGAGTTCAAAGGTGTGGA
cGAS R	GACTCAGCGGATTTCCTCGTG
STING F	CGGTTGATCTTACCAGGGCT
STING R	GGGGCAGCATATCTCGGAAT
Bax F	GACAGGGGCCTTTTTGCTACA
Bax R	CACGTCAGCAATCATCCTCTGC
NLRP3 F	ACCTCAACAGTCGCTACACG
NLRP3 R	ATGGTTTTCCCGATGCC
IRF3 F	GGAAAGAAGTGTTGCGGTTAG
IRF3 R	GGCTTGGCAGTTGTTGAGA
TBK1 F	CAAGAACTTATCTACGAAGGACG
TBK1 R	TGGATGTATTTTAGGGAGGGA
bcl-2 F	AGGATTGTGGCCTTCTTTGA
bcl-2 R	ACAAAGGCATCCCAGCC

### Western blotting assay

Tissues were lysed with cell lysate for 30 min. The lysate was transferred to a 1.5 mL centrifuge tube for centrifugation at 12000 r/min at 4° C for 5 min. The total protein concentration of the supernatant was determined by BCA method. After 15%SDS-PAGE electrophoresis separation, proteins were transferred to PVDF membrane, which was sealed with 5% skim milk powder and shaken at room temperature for 1.5 h. The PVDF membrane was removed and rinsed with TBST solution for 3 times, which was placed in a clean dish. cGAS (1:1000, ab252416, Abcam, Cambridge, UK), STING (1:1000, AF7916, Affinity, Melbourne, Australia), TBK1 (1:1000, DF7026, Affinity, Melbourne, Australia), IRF3 (1:1000, DF6895, Affinity, Melbourne, Australia), NLRP3 (1:1000, DF7438, Affinity, Melbourne, Australia), Bax (1:1000, AF0120, Affinity, Melbourne, Australia), Bcl-2 (1:1000, AF6139, Affinity, Melbourne, Australia), and β-actin (1:1000, Affinity, Melbourne, Australia) primary antibodies were added overnight at 4° C. TBST solution was added to rinse for 3 times, and the second antibody was added and incubated at room temperature for 1.5h. PVDF membrane was removed and rinsed with TBST solution for 3 times, and bands were obtained after exposing to ECL solution, which were further quantified using the Image J software.

### Immunofluorescence

Slice samples were placed on APES slides. After dewaxed, dried, antigen repaired, target antibodies were added and immunofluorescence staining was performed. The nuclei were stained by DAPI. Confocal scanning fluorescence microscopy was used to observe the staining results, and 5 fields were randomly selected for each image.

### ELISA assay

In brief, the required lath was removed from the aluminum foil bag after balancing for 60min at room temperature, and the remaining lath was sealed using a self-sealing bag in 4° C; After 50 μL adding different concentrations of standard to each well and 50 μL test sample into each well; 50 μL biotin-labeled antibody was introduced, followed by incubating at 37° C for 30min. After discarding the liquid and 3 washes, 100 μL horseradish peroxidase (HRP) labeled detection antibody was added to each well of standard and sample wells. The reaction wells were then sealed with a sealing plate membrane and incubated at 37° C for 30min. After discarding the liquid and 3 washes, 50 μL substrate A and substrate B were added to each well, and incubated at 37° C for 15min in the dark. After 50μL adding stop solution to each well, and the OD value of each well was measured at 450nm wavelength within 15min using a microplate analyzer (WD-2102B, LIUYI, Beijing, China).

### Statistical analysis

Data in the present study were expressed as mean±SD and were analyzed using the GraphPad software. The one-way ANOVA method was utilized for the analysis and P<0.05 was considered a significant difference.

## RESULTS

### EA and knockdown of cGAS significantly alleviated the depression symptoms in mice

**Figure 1:**
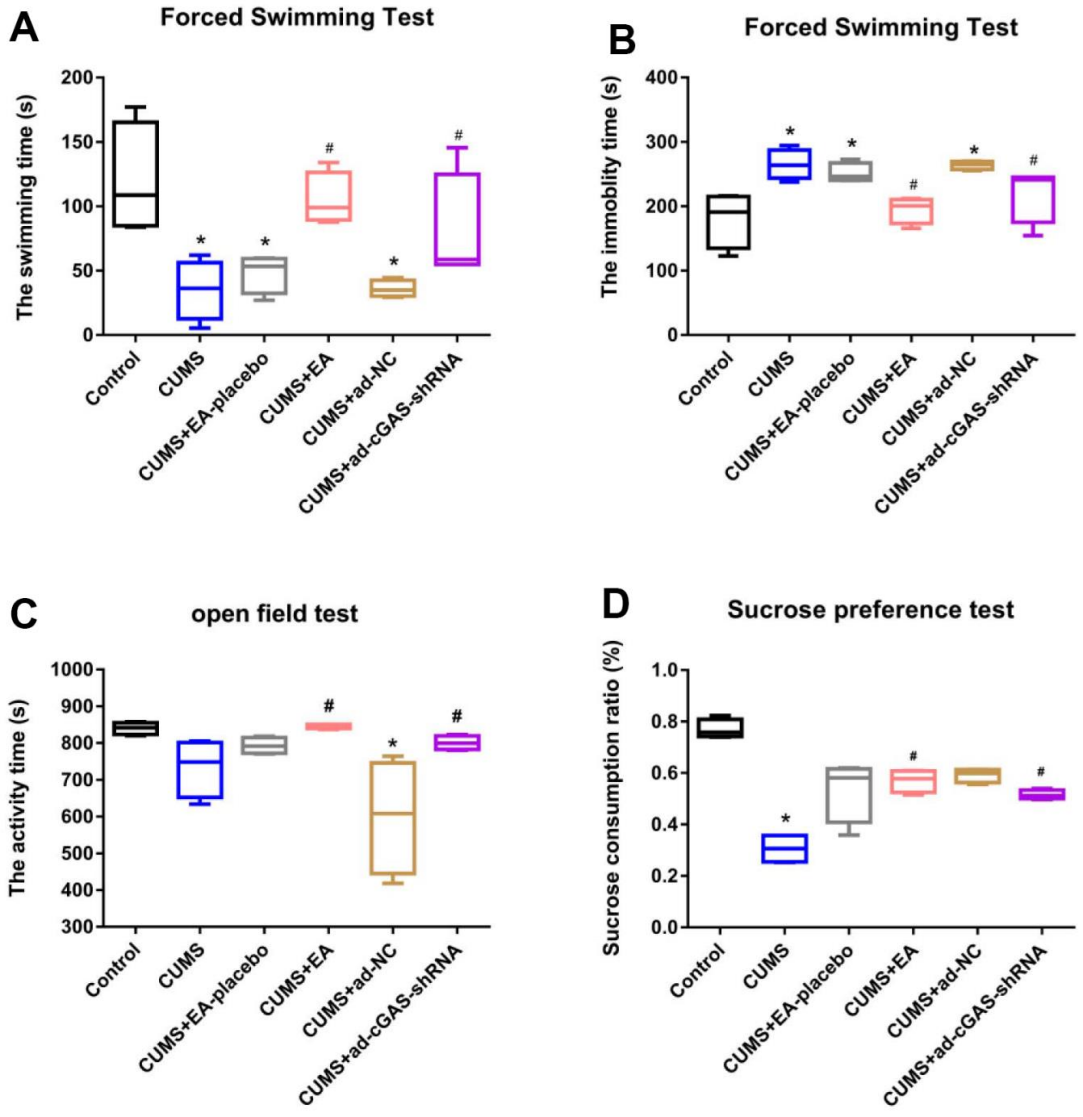
**The depression symptoms in mice were significantly alleviated by EA and knockdown of cGAS.** (**A**) Swimming time in the forced swimming test; (**B**) The immobility time in the forced swimming test; (**C**) The activity time in the open field test; (**D**) The sucrose consumption ratio in the sucrose preference test (*p<0.05 vs control, #p<0.05 vs CUMS).

Firstly, we determined the effect of EA intervention on depression-like behavior in depressed mice. The behavioral effects of EA on depressed mice were evaluated by field experiment, forced swimming test, and sucrose preference test at 28 days after CUMS modeling. In the forced swimming test (Figure 1A, 1B), compared to the control group, the forced swimming time of mice in the CUMS group was significantly reduced, and the floating time was greatly increased, which were dramatically reversed by the treatment of EA or the transfection of shRNA-cGAS. As shown in Figure 1C, significantly declined activity time was observed in the CUMS group, which was greatly elevated in the CUMS+EA and CUMS+ ad-cGAS-shRNA groups. In the sucrose preference test, the sucrose preference of mice in the CUMS group was significantly suppressed, which was greatly activated in the CUMS+EA and CUMS+ ad-cGAS-shRNA groups (*p<0.05 vs control, #p<0.05 vs CUMS). These results confirmed the anti-depressant effect of EA in a mouse model of depression, which might be related to the expression of cGAS.

### EA and knockdown of cGAS mitigated the pathological changes in the hippocampus of depressed mice

**Figure 2:**
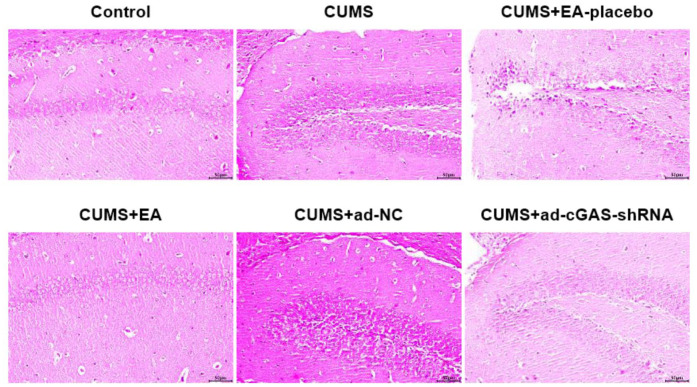
The impact of EA and knockdown of cGAS on the pathological state in the hippocampus was evaluated by HE staining assay.

HE staining was used to analyze the effect of EA intervention and cGAS interference on the pathological damage of hippocampus in depressed mice. As shown in Figure 2, in the CUMS group, the hippocampal neurons were significantly damaged, including cell swelling, nuclear shrinkage, and vacuole. In the CUMS+EA-placebo group, the hippocampal neurons were severely damaged, while in CUMS+EA and CUMS+ ad-cGAS-shRNA groups, the damaged hippocampal neurons were repaired, and the cell edema and vacuole were alleviated. These data revealed that EA and knockdown of cGAS effectively reversed the pathological damage in the hippocampal tissue in mice induced by CUMS modeling.

### The impact of EA and knockdown of cGAS on the expression level of apoptosis related proteins and proteins in the cGAS/STING signaling

**Figure 3:**
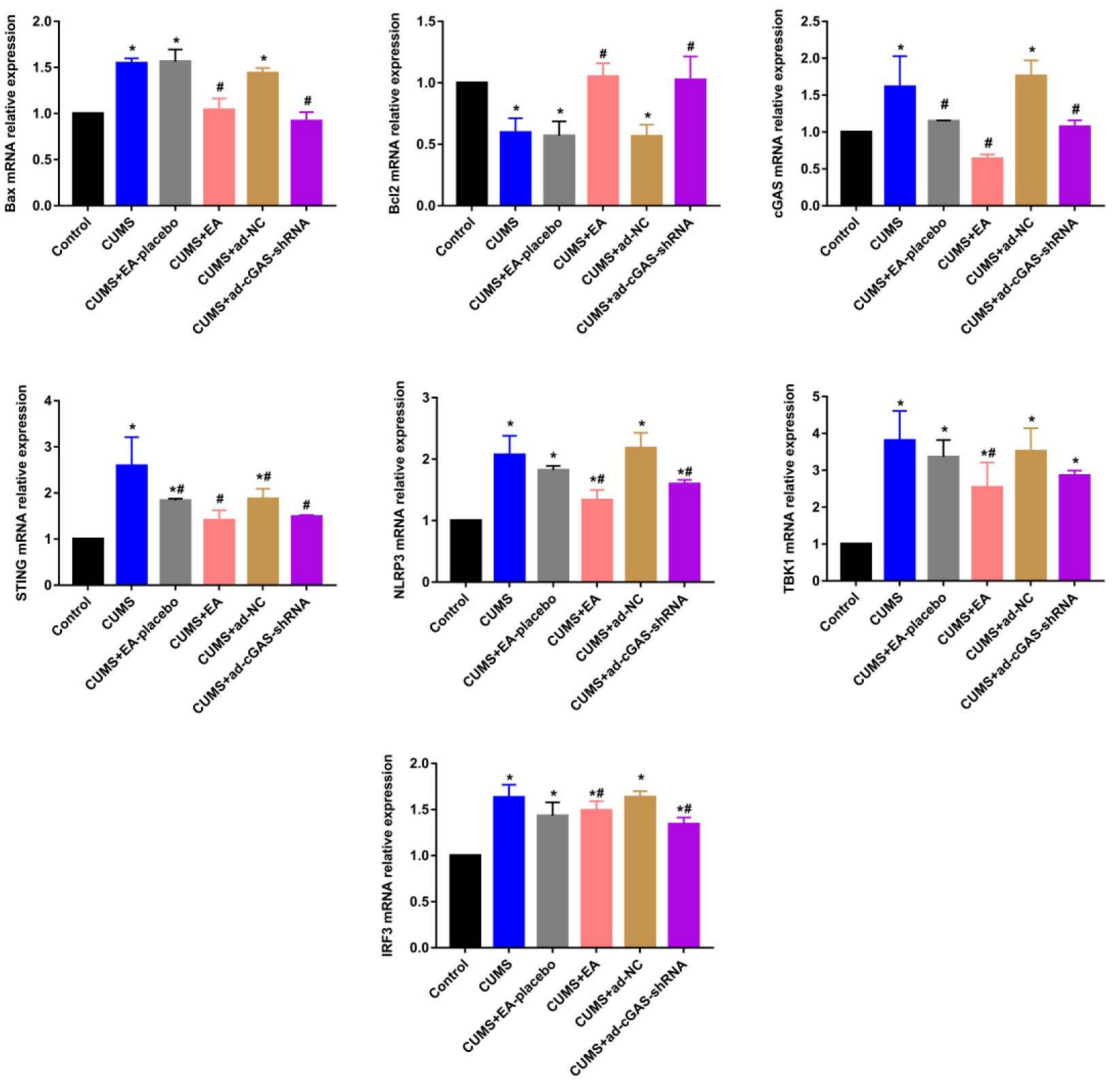
The impact of EA and knockdown of cGAS on the expression level of Bax, Bcl-2, cGAS, STING, TBK1, IRF3, and NLRP3 was determined by RT-PCR (*p<0.05 vs control, #p<0.05 vs CUMS).

**Figure 4:**
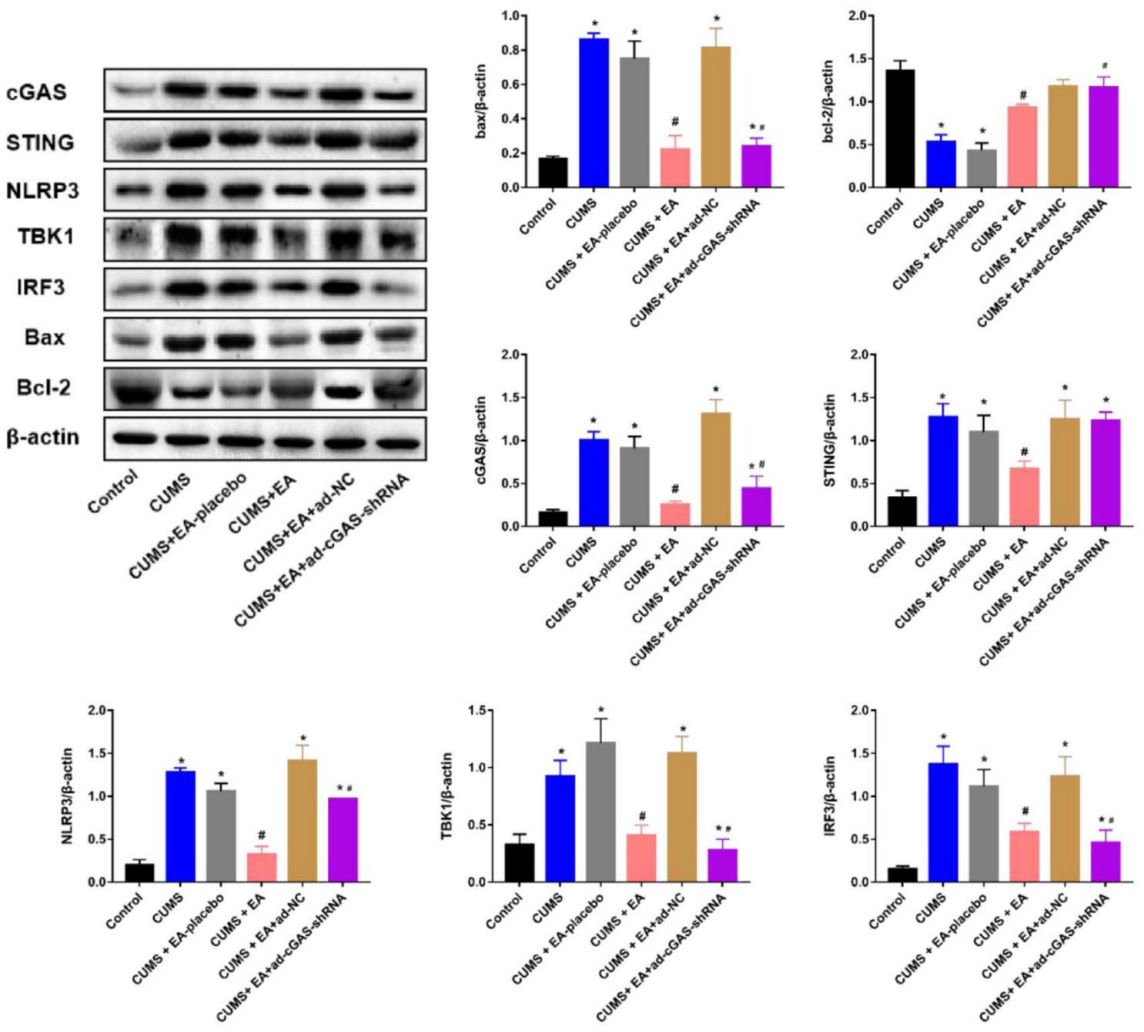
The impact of EA and knockdown of cGAS on the expression level of Bax, Bcl-2, cGAS, STING, TBK1, IRF3, and NLRP3 was determined by Western blotting (*p<0.05 vs control, #p<0.05 vs CUMS).

As shown in Figures 3, 4, the expression level of Bax in the CUMS group was significantly elevated, while the expression level of Bcl-2 was greatly reduced, which were dramatically reversed in the CUMS+EA and CUMS+ ad-cGAS-shRNA groups (*p<0.05 vs control, #p<0.05 vs CUMS). There were significant differences in mRNA and protein levels of cGAS, STING, NLRP3, TBK1 and IRF3 in hippocampus among all groups. The mRNA and protein expression levels of cGAS, STING, NLRP3, TBK1 and IRF3 in CUMS group were significantly higher than those in control group, which were dramatically repressed in the CUMS+EA and CUMS+ ad-cGAS-shRNA groups (*p<0.05 vs control, #p<0.05 vs CUMS). These results suggested that EA intervention and cGAS interference might exert the antidepressant property by regulating the expressions of apoptosis-related proteins and cGAS/STING pathway.

### The impact of EA and knockdown of cGAS on activation of microglia in depressed mice

**Figure 5:**
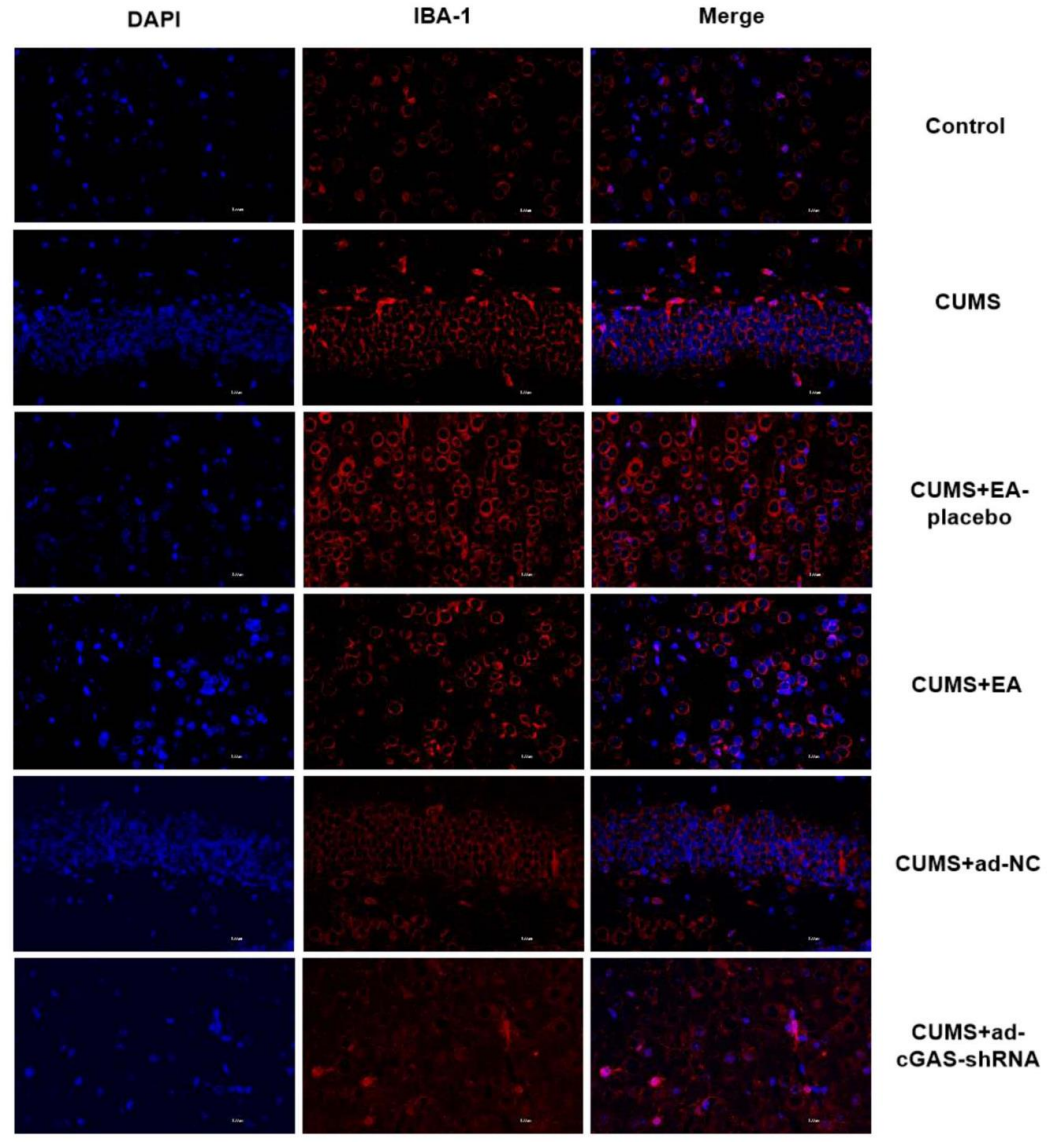
**EA and knockdown of cGAS significantly inhibited the activation of microglia in depressed mice.** The expression of IBA-1 in the hippocampus was measured using the immunofluorescence assay.

Microglia are common immunodeficient ganglion cells located in the central nervous system, which are associated with the progression of various neurological diseases. In the present study, the expression of IBA-1 in the hippocampus was detected by immunofluorescence to determine the activation of microglia. As shown in Figure 5, compared to control, the expression level of IBA-1 in the hippocampus of CUMS group was significantly upregulated, which was dramatically declined in the CUMS+EA and CUMS+ ad-cGAS-shRNA groups (*p<0.05 vs control, #p<0.05 vs CUMS). These results showed that CUMS stimulation activated microglia in the hippocampus of mice, which were dramatically repressed by EA and cGAS interference, suggesting that the therapeutic effect of EA and cGAS interference might be associated with the inhibition of microglia activation.

### The impact of EA and knockdown of cGAS on the concentration of monoamine in the hippocampus of depressed mice

**Figure 6:**
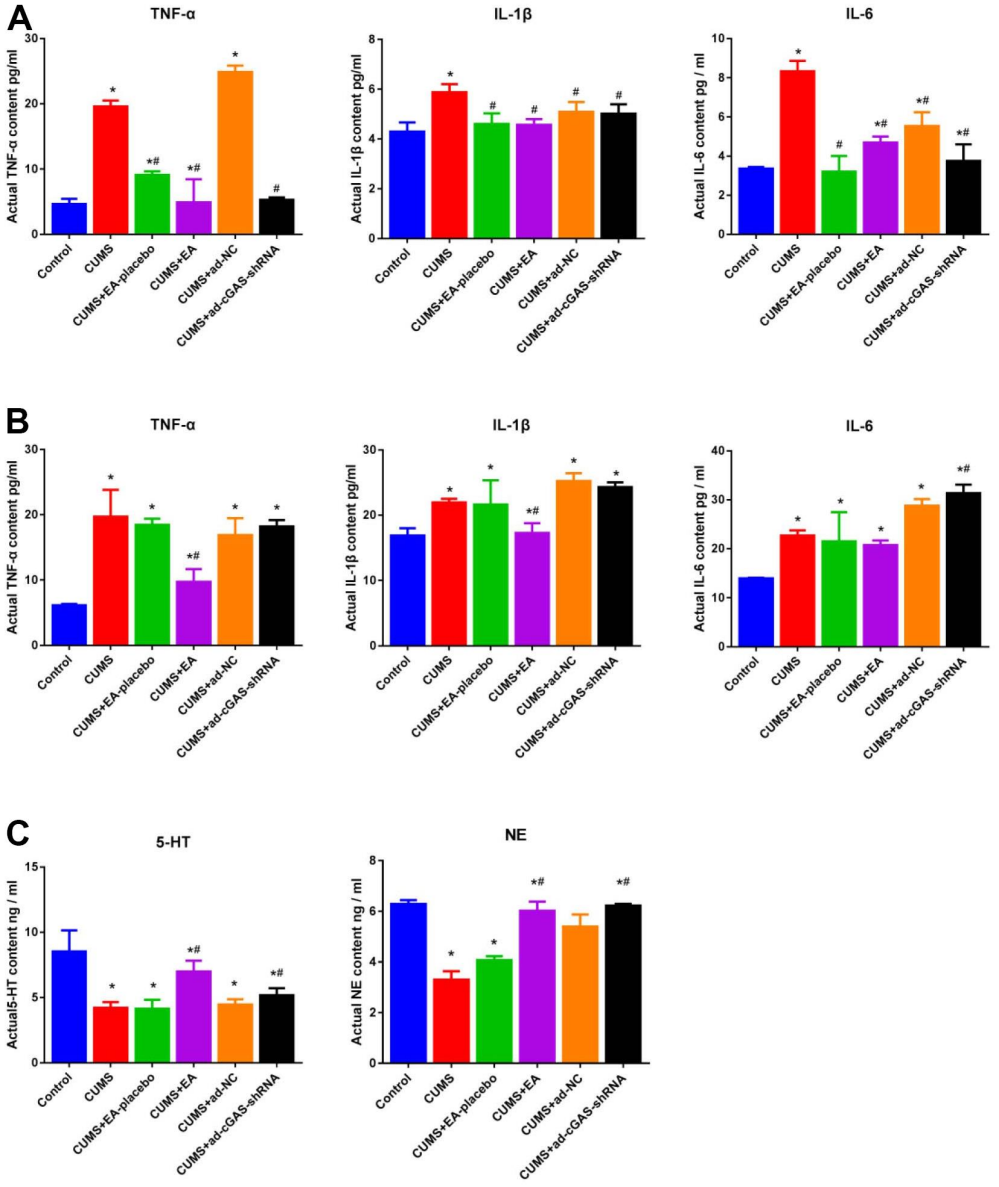
**The impact of EA and knockdown of cGAS on the production of inflammatory factors and monoamines in depressed mice.** (**A**) The concentration of TNF-α, IL-1β, and IL-6 in the serum was measured by ELISA. (**B**) The concentration of TNF-α, IL-1β, and IL-6 in the hippocampus was measured by ELISA. (**C**) The release of 5-HT and NE in the hippocampus was determined by ELISA (*p<0.05 vs control, #p<0.05 vs CUMS).

Inflammatory factors, 5-HT, and NE are closely related to the development of depression. In the present study, the concentration of inflammatory factors (TNF-α, IL-1β and IL-6) in serum and hippocampal tissues of mice were detected and the contents of 5-HT and NE in hippocampal tissues were detected using ELISA. As shown in Figure 6, compared to control, the concentration of TNF-α, IL-1β, and IL-6 in serum and hippocampus was greatly promoted in the CUMS group, which was dramatically repressed in the CUMS+EA and CUMS+ ad-cGAS-shRNA groups. Furthermore, the declined concentration of 5-HT and HE in the CUMS group was significantly increased in the CUMS+EA and CUMS+ ad-cGAS-shRNA groups (*p<0.05 vs control, #p<0.05 vs CUMS). The results revealed that CUMS stimulation significantly increased the levels of inflammatory factors in serum and hippocampus of mice, and decreased the levels of monoamine in the hippocampus, which were dramatically alleviated by EA or knockdown of cGAS.

## DISCUSSION

In the present study, the depression model was successfully established in mice using the CUMS method, which was utilized to determine the therapeutic effect of EA on the depressive behavior and neural damage, and to explore the potential mechanism. The cGAS-STING signaling pathway has been shown to be involved in the progression of various diseases. However, its role in depression remains uncertain. Our results suggested that EA treatment improved CUMS-induced depressed-like behavior and alleviated neurological damage in mice, which was possibly mediated by inhibition of the cGAS-STING pathway.

CUMS, as a risk factor for mental illness, has been widely used in animal models of mood disorders. CUMS model simulates stress-induced depression [24] and has been widely used in preclinical studies. Animal studies have shown that behavioral symptoms of depression, such as reduced autonomic activity time, increased resting time, and reduced sucrose consumption, can be induced by CUMS [25, 26]. Consistently, our results showed that after 28 days of CUMS stimulation, swimming time, resting time, activity time, and sucrose consumption were significantly reduced, while EA treatment reversed these behavioral changes, suggesting that EA showed a favorable antidepressant effect, which was consistent with results reported previously [27, 28]. In addition, the downregulation of cGAS reversed these changes, and the downregulation of cGAS showed an antidepressant effect.

One of the major brain regions in which EA improves depressed behavior in animals is the hippocampus, and some of the mechanisms have been confirmed, such as inhibition of neuroinflammation and neurotransmitter upregulation [29, 30]. Microglial abnormalities are associated with a variety of brain diseases, including depression [31]. Extensive activation of microglia can be induced following various stimuli, which will be polarized into M1 phenotype and secrete pro-inflammatory cytokines, such as TNF-α, IL-1β, and IL-6, to further aggravate inflammation [32]. CUMS stimulation has been reported to activate microglia in the brain of mice, accompanied by the elevated release of pro-inflammatory factors, including TNF-α, IL-1β, and IL-6 [33] and declined concentration of neurotransmitters, such as 5-HT and NE, in the brain tissues [34]. Our results showed that EA improved the pathological brain damage in CUMS mice, suppressed microglia activation, and reduced the levels of TNF-α, IL-1β, and IL-6 accompanied by elevated levels of 5-HT and NE, which were consistent with the report mentioned above. Downregulation of cGAS reduced microglia activation and the levels of TNF-α, IL-1β, and IL-6, which increased the levels of 5-HT and NE. Therefore, EA and cGAS downregulation suppressed microglia activation, inhibited pro-inflammatory factor expression, and increased neurotransmitter expression.

cGAS-STING signaling has been reported to play an important role in microbial infection, chronic inflammation, tumor progression, and organ degeneration [35–37]. However, the role of cGAS-STING signaling axis in depression has not been clarified. NLRP3 inflammasome is an iso-oligomeric protein complex involved in multiple validation-related disease progression [38]. TBK1 and IRF3 are downstream proteins of STING pathway [39]. Furthermore, the CGAS-STING axis promotes LPS-induced acute lung injury by regulating NLRP3 inflammasome in macrophages [40]. Our results showed that CUMS stimulation increased the expression of cGAS, STING, NLRP3, TBK1, and IRF3 in the CGAS-STING pathway in depressed mice, while EA and cGAS intervention reversed these changes, suggesting that the effect of EA on depression might be mediated by inhibiting the CGAS-STING pathway.

Collectively, the present study investigated the role of depression in CGAS-STING signaling in mice. Our results showed that EA and cGAS interference significantly alleviated depressive-like behavior of CUMS mice, accompanied by alleviated pathological damage of hippocampus, inhibited pro-inflammatory cytokines, promoted monoamine neurotransmitters production, and inactivated microglia. Furthermore, EA and cGAS knockdown significantly reduced the expression of cGAS, STING, TBK1, IRF3, and NLRP3, suggesting that the mechanism of EA’s anti-depressive effect may be mediated by inhibiting the cGAS-STING signaling axis.

The present study has some limitations. Firstly, only one time point was selected for behavioral tests in this study according to some references, and the evidence is slightly insufficient. Secondly, the experiment was not able to activate the cGAS-STING pathway during EA to directly prove that EA’s anti-depressive effect is mediated by inhibiting the cGAS-STING pathway. In addition, mouse behaviors and responses cannot fully reflect human depression. The present study only explored the improvement effect of EA on mouse depression symptoms at the animal level, and its possible mechanism. If combined with clinical samples and detection of related molecules, the conclusion of this article will be more persuasive. However, results of the present study have initially shown that EA can improve core depressive symptoms in mice, and its mechanism may be to inhibit microglia activation and neuroinflammation by inhibiting the cGAS-STING-NLRP3 signaling pathway. The cGAS-STING signaling axis may be a promising target for the treatment of depression.
